# Impact of viral disease hypophagia on pig jejunal function and integrity

**DOI:** 10.1371/journal.pone.0227265

**Published:** 2020-01-07

**Authors:** Emma T. Helm, Shelby M. Curry, Carson M. De Mille, Wesley P. Schweer, Eric R. Burrough, Nicholas K. Gabler

**Affiliations:** 1 Department of Animal Science, Iowa State University, Ames, Iowa, United States of America; 2 Department of Veterinary Diagnostic and Production Animal Medicine, Iowa State University, Ames, Iowa, United States of America; University of Kentucky, UNITED STATES

## Abstract

Pathogen challenges are often accompanied by reductions in feed intake, making it difficult to differentiate impacts of reduced feed intake from impacts of pathogen on various response parameters. Therefore, the objective of this study was to determine the impact of Porcine Reproductive and Respiratory Syndrome virus (**PRRSV**) and feed intake on parameters of jejunal function and integrity in growing pigs. Twenty-four pigs (11.34 ± 1.54 kg BW) were randomly selected and allotted to 1 of 3 treatments (*n* = 8 pigs/treatment): 1) PRRSV naïve, ad libitum fed (**Ad**), 2) PRRSV-inoculated, ad libitum fed (**PRRS+**), and 3) PRRSV naïve, pair-fed to the PRRS+ pigs’ daily feed intake (**PF**). At 17 days post inoculation, all pigs were euthanized and the jejunum was collected for analysis. At days post inoculation 17, PRRS+ and PF pigs had decreased (*P* < 0.05) transepithelial resistance compared with Ad pigs; whereas fluorescein isothiocyanate-dextran 4 kDa permeability was not different among treatments. Active glucose transport was increased (*P* < 0.05) in PRRS+ and PF pigs compared with Ad pigs. Brush border carbohydrase activity was reduced in PRRS+ pigs compared with PF pigs for lactase (55%; *P* = 0.015), sucrase (37%; *P* = 0.002), and maltase (30%; *P* = 0.015). For all three carbohydrases, Ad pigs had activities intermediate that of PRRS+ and PF pigs. The mRNA abundance of the tight junction proteins claudin 2, claudin 3, claudin 4, occludin, and zonula occludens-1 were reduced in PRRS+ pigs compared with Ad pigs; however, neither the total protein abundance nor the cellular compartmentalization of these tight junction proteins differed among treatments. Taken together, this study demonstrates that the changes that occur to intestinal epithelium structure, function, and integrity during a systemic PRRSV challenge can be partially explained by reductions in feed intake. Further, long term adaptation to PRRSV challenge and caloric restriction does reduce intestinal transepithelial resistance but does not appear to reduce the integrity of tight junction protein complexes.

## Introduction

Integrity and function of the small intestinal epithelium is crucial for optimizing the wellbeing, lean accretion, and feed efficiency of growing pigs [1]. In a production setting, it is inevitable that the pig’s small intestinal epithelium will be exposed to a variety of toxins and pathogens, thus it must provide a barrier to protect the host from injury and infection. Concurrently, the small intestine is responsible for the majority of nutrient digestion and absorption, which is vital for maintenance and growth. As such, the gastrointestinal tract has developed a sophisticated system of digestive enzymes, transporters, and barriers. The barrier system primarily relies on integrity of tight junction complexes, which consist of several transmembrane proteins including claudins and occludin as well as intracellular proteins such as zonula occludens-1 (**ZO-1**), which scaffold transmembrane proteins to the cytoskeleton [2]. This system works to simultaneously prevent pathogen entry whilst allowing for efficient uptake of dietary nutrients and electrolytes. Stressors that impact the intestine such that barrier integrity and digestive function are hindered have far reaching consequences on the host that can result in both suboptimal health and growth performance.

Reductions in intestinal integrity and function have been documented under a variety of stressors, including heat stress [3, 4], weaning stress [5, 6], and enteric disease challenges such as with Porcine Epidemic Diarrhea Virus [**PEDV**; 7, 8, 9]. However, reduced nutrient intake is often reported in accompaniment of these stressors, which alone can modulate intestinal integrity and function [10–12]. Our group has previously reported that stress induced hypophagia, or reduced voluntary feed intake, may explain the majority of changes reported in intestinal integrity and function in pigs during heat stress [4], however if the same holds for pigs during disease challenge remains unclear.

Reduced nutrient intake under health compromised conditions, known as disease anorexia or hypophagia, is an aspect of sickness behavior conserved across nearly all animal species [13–15]. In pigs, disease anorexia is a major reason for the loss in lean tissue growth observed during both viral [16–18] and bacterial [19–21] challenges. One viral disease characterized by a marked reduction in feed intake is Porcine Reproductive and Respiratory Syndrome, which remains one of the most problematic swine diseases worldwide [22]. The causative pathogenic agent for this disease is the double-stranded RNA Porcine Reproductive and Respiratory Syndrome Virus (**PRRSV**), which induces respiratory disease in growing pigs accompanied by a reduction in both feed intake and growth performance [18, 23] that can even result in weight stasis or loss [16].

In addition to reducing growth via reductions in feed intake, activating the immune system to respond to a viral challenge is energetically demanding [24]. Fueling inflammatory responses, cellular migration, respiratory burst, fever, and proliferation and maintenance of immune cell populations likely demands a reallocation of dietary nutrients and tissue reserves [25, 26]. As such, it is possible mounting an immune defense competes with other host maintenance and production processes for resources, although the extent to which this occurs in pigs is unclear. We have previously demonstrated that a reduction in feed intake accounts for a significant portion of the alterations in skeletal muscle accretion and metabolism during PRRSV challenge [16] and thus aimed to investigate if the same held true for other body systems, such as the intestine. The gastrointestinal tract is highly metabolic, accounting for 20–30% of the major basal metabolic rate [27]. Therefore, although the intestine is not the primary system affected by PRRSV, alterations to its structure and function may still occur to spare nutrients for the immune response. Thus, the objectives of this study were to evaluate the direct effects of PRRSV challenge versus the indirect effects of caloric restriction on intestinal barrier integrity and function.

## Materials and methods

All animal procedures were approved by the Iowa State University Institutional Animal Care and Use Committee (IACUC protocol # 8-15-8074-S) and adhered to the ethical and humane use of animals for research.

### Experimental design, inoculations, and energy calculations

This project was designed as part of a larger project previously described [16]. For the current study, 24 gilts (Genetiporc 6.0 × Genetiporc F25, PIC, Inc., Hendersonville, TN) confirmed seronegative by ELISA for PRRSV antibodies and viremia by PCR were weighed and assigned to blocks of 3 pigs/block based on initial body weight (**BW**; 11.34 ± 1.54 kg BW). This resulted in 3 treatment groups: 1) PRRSV naïve, Ad libitum fed (**Ad**, *n* = 8), 2) PRRSV inoculated, ad libitum fed (**PRRS+**, *n* = 8), and 3) PRRSV naïve, pair-fed (**PF**, *n* = 8) to mirror nutrient intake of PRRS+ pigs. All pigs were housed individually at the Iowa State Livestock Infectious Disease Isolation Facility (Biosafety Level 2), with Ad and PF pigs housed in a separate room from PRRS+ pigs to prevent contamination. For the duration of the study, pigs were fed a common corn-soybean-meal based diet that met or exceeded their energy and nutrient requirements (NRC, 2012). Briefly, this diet was formulated to contain 3,332 kcal/kg metabolizable energy and a standardized ileal digestible lysine of 1.10%.

On days post inoculation (**dpi**) 0, PRRS+ pigs were inoculated with an ORF5 RFLP_1-3-4 clinical isolate of PRRSV via 1 mL intramuscular injection and 1 mL intranasal inoculation (10^6^ genomic units per mL). The Ad and PF pigs were administered the same volume of saline intramuscularly and intranasally as a sham. At and beyond dpi 0, PF pigs were fed daily to the previous days voluntary feed intake of the PRRS+ pig in its respective block. To implement pair feeding, voluntary feed consumption of each PRRS+ pig was recorded every morning by weighing feeders and calculating feed disappearance. That amount of feed was then given to the PF pig in its respective block the following morning. Over the 17 day challenge period, start and end body weights, average daily gain (**ADG**), average daily feed intake (**ADFI**), and feed efficiency (Gain:Feed; **G:F**) were recorded and calculated.

### Sample collection

On dpi 17, all pigs were euthanized via captive bolt followed by exsanguination for tissue collection. This timepoint was chosen as it would likely coincide with seroconversion, capturing an energy demanding time for the immune response and remaining in a period in which peak growth performance impact would be occurring [28, 29]. Immediately prior to euthanasia, blood samples were collected from all pigs and were submitted to the Iowa State Veterinary Diagnostic Laboratory to measure PRRSV viral titers and antibody levels via PCR and ELISA assays routine to the laboratory. This data confirmed PRRS+ pigs were successfully inoculated and that Ad and PF pigs remained naïve to PRRSV, as has been presented elsewhere [16]. Jejunum sections were collected 3 m proximal to the ileal-cecal junction, flushed with Krebs-Henseleit buffer (containing 25 mM NaHCO_3_, 120 mM NaCl, 1 mM MgSO_4_, 6.3 mM KCl, 2 mM CaCl_2_, and 0.32 mM NaH_2_PO_4_), and placed into continuously aerated containers containing Krebs-Henseleit buffer. These were transported to the lab to be mounted in modified Ussing chambers for electrophysiological measurements. Additional adjacent jejunum sections were collected and used to: 1) collect mucosal scrapings that were snap-frozen in liquid nitrogen and stored at -80°C prior to analysis and 2) collect in 10% neutral buffered formalin for fixation. Fixed samples were switched to 70% ethanol after approximately 24 h of fixation and were later submitted to the Iowa State Veterinary Diagnostic Laboratory to be paraffin-embedded, sectioned, and mounted on slides for routine histological staining and evaluation.

### *Ex vivo* assessment of jejunal barrier function and integrity

Fresh jejunum sections were mounted in modified Ussing chambers within approximately 1 to 1.5 h of euthanasia. Modified Ussing chambers were assembled and electrophysiological and fluorescein isothiocyanate-dextran 4 kDa (**FD4**) macromolecule permeability measurements were collected as previously described [30]. Estimates of nutrient transport are calculated as the change in current (μA) after glucose and glutamine addition. A fluorescent plate reader (Cytation 5 Hybrid Multi-Mode Reader, BioTek Instruments Inc., Winooski, VT) was used to determine mucosal to serosal flux changes in relative fluorescence of FD4 in the serosal samples from 0 to 60 minutes after FD4 addition at 485 and 520 nm excitation and emission wavelengths, respectively. Permeability coefficients for FD4 flux were calculated as described by Pearce et al., (2013).

### Brush border enzyme activities

Jejunal Na^+^/K^+^ ATPase activity was determined in protein extracted from frozen jejunal mucosal scrapings. Scrapings (0.5 g) were homogenized in sucrose buffer (50 mM sucrose, 1 mM Na_2_EDTA, and 20 mM Tris base; pH 7.4) and centrifuged at 1000 × *g* for 10 minutes at 4⁰ C. The supernatant was collected, and protein concentration was determined via a Pierce bicinchoninic acid (**BCA**) assay (ThermoFisher Scientific, Waltham, MA). The protein extract was then separated into 4 aliquots: 2 for blanks and 2 for ouabain incubations. Proteins with either water (blanks) or 20 mM ouabain were pre-incubated at 37°C with Na^+^/K^+^ ATPase reaction buffer (2000 mM NaCl, 100 mM KCl, 50 mM MgCl_2_, and 250 mM HEPES, pH 7.0) for 15 minutes. The reaction was then initiated by the addition of fresh 105 mM ATP and samples were incubated at 37°C for 45 minutes. The reaction was then terminated by the addition of 50% trichloroacetic acid. Samples were centrifuged at 1,500 × *g* for 10 minutes and the resulting supernatant was analyzed for the presence of inorganic phosphate using the Molybdovanadate method [31] and assessed in duplicate at a wavelength of 400 nm (Cytation 5 Hybrid Multi-Mode Reader, Biotek Instruments Inc., Winooski, VT). Specific Na^+^/K^+^ ATPase activity was determined by the difference in inorganic phosphate (P_i_) production from ATP in the presence or absence of ouabain (specific Na^+^/K^+^ ATPase inhibitor). Activities are presented as liberated inorganic phosphate (μmol) per mg protein per hour.

Activities of brush border maltase, sucrase, and lactase were analyzed in protein extracted from frozen jejunum mucosal scrapings. For protein extraction, mucosal scrapings (0.5 g) were weighed out into 4 ml of PBS buffer containing 1% Triton X-100 and 0.1% protease inhibitor cocktail, homogenized, and then centrifuged at 2,000 × *g* for 10 minutes at 4 ⁰C. The supernatant was collected, protein concentrations were determined via BCA, and extracted protein was stored at -80⁰C until analyzed. Activities of maltase, sucrase, and lactase were analyzed using a modified method previously described [3, 32]. Activity was determined by the amount of glucose liberated from each sample after correcting for basal glucose levels, measured using the glucose oxidase kit (Sigma, St. Louis, MO) on plates read at 540 nm (Cytation 5 microplate reader, Bio-Tek, Winooski, VT). Sample activities are presented as liberated glucose (mM) per gram protein per minute.

Activity of brush border aminopeptidase was determined in protein extracted in PBS extraction buffer as described above. L-alanine aminopeptidase activity was analyzed using a modified method of Roncari and Zuber [33], using 1-alanine-4-nitroaniline as a substrate to examine the release of 4-nitroaniline. Sample activities are presented as liberated 4-nitroaniline (μM) per mg protein per minute.

### Western blot

Protein abundance of occludin, claudin 4, phosphorylated adenosine 5’ monophosphate kinase α (**AMPK**), and total AMPK were evaluated via western blot to examine markers of both intestinal integrity (occludin, claudin 4) and energy sensing (AMPK). Protein was extracted from jejunum mucosal scrapings into HEPES cell lysis buffer (50 mM HEPES, 150 mM NaCl, 50 mM NaF, 2 mM EDTA, 1% Triton X-100, 0.1% protease inhibitor cocktail, 5% glycerol, and 0.1% SDS) and protein concentrations determined via BCA. Equal protein amounts (20 μg per lane) were separated by SDS polyacrylamide gel electrophoresis and Western blots were run as previously described [16]. Antibodies used were: occludin (Invitrogen #33–1500), claudin 4 (Life Technologies #329400), total AMPK (Cell Signaling Technology #2532), and AMPKα Thr^172^ (**pAMPK**; Cell Signaling Technology #2535).

### Quantitative polymerase chain reaction

Total mRNA was extracted from frozen jejunum scrapings utilizing a Direct-zol RNA Miniprep Kit (Zymo Research, Irvine, CA). Quantity and purity of extracted mRNA was determined spectrophotometrically using a Cytation 5 Hybrid Multi-Mode Reader (BioTek Instruments Inc., Winooski, VT). All samples had a 260/280 ratio of at least 1.8. Five-hundred nanograms of extracted mRNA was transcribed using a commercially available kit (Quantitect reverse transcription kit, Qiagen Inc., Valencia, CA) and resulting cDNA was utilized for quantitative real-time PCR using an iQ5 Optical System (Bio-Rad Laboratories, Inc., Hercules, CA) and iQ SYBR Green Supermix (Bio-Rad Laboratories, Inc., Hercules, CA). The mRNA abundance values were normalized to a reference gene (*ACTB*) and Ad pigs according to the 2^-ΔΔCt^ method. Gene symbols and primer sequences are listed in S1 Table.

### Histology and immunohistochemistry

Jejunum sections were stained with hematoxylin and eosin to evaluate intestinal morphology. Images were taken at 20X magnification using a DP80 Olympus Camera mounted on an OLYMPUS BX 53/43 microscope (Olympus Scientific, Waltham, MA). Fifteen to twenty well orientated villus and crypt pairs were measured using OLYMPUS CellSens Dimension 1.16 software (Olympus Scientific, Waltham, MA).

To evaluate goblet cells, jejunum sections were deparaffinized and were incubated in Alcian blue (pH 2.5). Slide sections were bathed in Alcian blue for 5 minutes, rinsed with water, dehydrated, and then mounted. Using a DP80 Olympus Camera mounted on an OLYMPUS BX 53/43 microscope with a motorized stage, 2–3 images were taken at 20X magnification, to acquire at least 15 well-orientated villus and crypt pairs. Images were analyzed using HALO image analysis software (HALO^™^, Indica Labs, Inc., Corrales, NM). The HALO software output provided a count of Alcian blue positive cells per area, thus individual villi and their adjacent crypts were outlined as the region of interest, goblet cells were counted, and presented as number of goblet cells per 10,000 μm^2^_._

To further assess jejunal integrity, immunohistochemistry slides were prepared from embedded jejunal tissue. Commercially available primary antibodies for claudin 2 (Invitrogen #32–5600), claudin 4 (Invitrogen #32–9400), and ZO-1 (Invitrogen #40–2300) were used. Slide staining was performed as previously described [7]. Images were taken as described above and analyzed using HALO image analysis software (HALO^™^, Indica Labs, Inc., Corrales, NM). Individual villi and their adjacent crypts were outlined as the region of interest, only including the epithelial layer of tissue. As the proteins of interest are located at the membrane under normal conditions, stain intensity at the membrane and within the cytoplasm were both evaluated. These data were expressed as the average optical density of staining within the region of interest.

### RNA chromogenic in-situ hybridization

Visualization of mRNA transcripts were performed as according to the manufacturer’s instructions for RNAScope^®^ 2.5 (Advanced Cell Diagnostics, Hayward, CA, USA) using *Sus scrofa*-specific proprietary probe combinations for claudin 4, occludin, ZO-1, and AMPK (Advanced Cell Diagnostics, Hayward, CA, USA). Slides were then imaged at 40X magnification using a DP80 Olympus Camera mounted on an OLYMPUS BX 53/43 microscope. Three images per slide were taken to acquire approximately 8–9 well orientated villi per pig. These individual villi and their adjacent crypts were outlined as the region of interest. Images were analyzed using the RNA in-situ hibridization module of HALO image analysis software (HALO^™^, Indica Labs, Inc., Corrales, NM). The module identified chromogenic duplex signals (red or brown) and these signals were quantified. These data are presented as the mean number of mRNA transcripts per cell within the region of interest.

### Statistical analysis

The SAS 9.4 program (SAS Institute Inc., Cary, NC) was used for statistical analysis of all data. The following mixed model was fitted to all parameters:

$$Y_{ijk}=\mu +PRRSV_{i}+iBW_{j}+e_{ijk}$$

wherein Y_ijk_ = the phenotype measured on animal *k*; PRRSV_i_ = effect of treatment (fixed effect; Ad, PF, PRRS+); iBW_j_ = blocking factor in which pigs were placed into blocks of 3 pigs/block based on initial body weight (random effect); and e_ijk_ = error term of animal *k* subjected to treatment *i* in block *j*, e_ijk_ ~ N(0, σ_e_^2^). Least square means were determined using the LS means statement and differences in LS means were produced using the pdiff option. Histochemistry count data were evaluated using the GLIMMIX procedure and a Poisson distribution. Contrast statements were used to determine the effect of reduced nutrient supply (Ad vs others) and the effect of PRRSV additional to reduced nutrient supply (PF vs PRRS+). All data are presented as Least Squares means with a pooled standard error. Differences were considered significant when *P <* 0.05 and a tendency when 0.05 ≤ *P* ≤ 0.10.

## Results

### Growth performance

Initial body weights did not differ amongst treatments (*P* = 0.727; **Table 1**), however at dpi 17 PRRS+ and PF pigs had a 34% reduction in body weight when compared with Ad pigs (*P* < 0.001). Average daily gain was significantly reduced in both PF (76%; *P* < 0.001) and PRRS+ (104%, *P* < 0.001) pigs compared with Ad pigs (**Table 1**). Additionally, ADG was further reduced in PRRS+ (113%, *P* < 0.015) pigs compared with PF pigs. Average daily feed intake was reduced in both PF and PRRS+ (*P* < 0.001) pigs compared with Ad pigs. Consistent with experimental design and validating the pair-feeding model, ADFI did not differ between PF and PRRS+ pigs (*P* = 0.366). Gain:feed was reduced in PRRS+ and PF pigs compared with Ad pigs (*P* = 0.001). Gain:feed was additionally reduced in PRRS+ pigs compared with PF pigs (*P* = 0.006).

**Table 1:** Growth performance^1^.

	Treatment				*P*-value	
Item	Ad	PF	PRRS+	SEM	Ad vs. others	PF vs. PRRS+
Start BW, kg	12.7	12.4	13.3	0.733	0.907	0.435
End BW, kg	21.0	14.9	13.0	1.285	<0.001	0.123
ADG, kg/d	0.50	0.15	-0.02	0.047	<0.001	0.015
ADFI, kg/d	0.87	0.48	0.44	0.045	<0.001	0.366
Gain:Feed	0.57	0.29	-0.14	0.105	0.001	0.006

^1^ Pigs were either challenged with porcine respiratory and reproductive syndrome virus (PRRS+), PRRSV naïve and fed ad libitum (Ad), or PRRSV naïve and pair-fed to PRRS+ pigs intake (PF). Pigs were euthanized at days post inoculation (dpi) 17.

### Jejunum integrity, digestive enzyme activity, and nutrient absorption

Jejunum barrier integrity as assessed via transepithelial resistance was significantly reduced in PF and PRRS+ pigs compared with Ad pigs (40%, *P* < 0.001), but transepithelial resistance did not differ between PF and PRRS+ pigs (*P* = 0.679; **Table 2**). *Ex vivo* assessment of mucosal to serosal FD4 macromolecule permeability did not differ amongst treatments (*P* > 0.10).

**Table 2:** Jejunum integrity, digestive enzyme activity, and active nutrient absorption^1^.

	Treatment				*P*-value	
Item	Ad	PF	PRRS+	SEM	Ad vs. others	PF vs. PRRS+
TER^2^	96.38	55.64	59.80	7.083	<0.001	0.679
FD4 flux^3^	26.18	39.49	42.49	8.031	0.114	0.783
Glucose, μA^4^	19.18	41.25	76.06	10.28	0.003	0.014
Glutamine, μA^4^	1.59	9.01	14.83	3.171	0.002	0.069
Na^+^-K^+^ ATPase^5^	1.93	0.95	1.28	0.334	0.048	0.479
Lactase^6^	7.34	11.12	5.01	1.657	0.678	0.006
Sucrase^6^	19.84	24.94	15.67	2.485	0.819	0.001
Maltase^6^	56.01	66.07	46.27	5.847	0.975	0.006
Aminopeptidase^7^	4351	4737	4087	452.3	0.914	0.321

^1^ Pigs were either challenged with porcine respiratory and reproductive syndrome virus (PRRS+), PRRSV naïve and fed ad libitum (Ad), or PRRSV naïve and pair-fed to PRRS+ pigs intake (PF). Pigs were euthanized at days post inoculation (dpi) 17.

^2^TER = transepithelial resistance, Ω × cm^2^

^3^Macromolecule (FD4) permeability, ug/mL/min/cm^2^

^4^Active absorption calculated by subtracting μA before substrate (glucose or glutamine) from μA after substrate addition.

^5^μmol liberated inorganic P/h/mg protein

^6^μM liberated 4-nitroaniline/min/mg protein

^7^μmol liberated glucose/min/g protein

Active transport of glucose was greater in PRRS+ and PF pigs when compared with Ad pigs (*P* = 0.003; **Table 2**) and was greater in PRRS+ pigs when compared with PF pigs (*P* = 0.014). Active glutamine transport was greater in PRRS+ and PF pigs compared with Ad pigs (*P* = 0.002). Active glutamine transport tended to be greater in PRRS+ pigs compared with PF pigs (*P* = 0.069).

Activity of the Na+/K+ ATPase was reduced in PF and PRRS+ pigs when compared with Ad pigs (*P* = 0.048; **Table 2**) and did not differ between PRRS+ and PF pigs. Activity of the carbohydrases was altered by both PRRSV and nutrient restriction. Interestingly, activity was reduced in PRRS+ pigs compared with PF pigs for lactase (55%; *P* = 0.015), sucrase (37%; *P* = 0.002), and maltase (30%; *P* = 0.015). For all three carbohydrases, Ad pigs had activities intermediate that of PRRS+ and PF pigs. Activity of aminopeptidase was not different amongst treatments (*P* > 0.10).

### Morphology and goblet cell count

Villus height was reduced in PRRS+ and PF pigs compared with Ad pigs (*P* = 0.003) and was additionally reduced in PRRS+ pigs compared with PF pigs (*P* = 0.036; **Table 3**). Crypt depth was reduced in PRRS+ and PF pigs compared with Ad pigs (*P* = 0.017), however crypt depth did not differ between PRRS+ and PF pigs. The ratio of villus height to crypt depth was increased in PRRS+ pigs compared with PF pigs (*P* < 0.001). Abundance of goblet cells did not differ amongst treatments (*P* > 0.10).

**Table 3:** Jejunum morphology and goblet cell counts^1^.

	Treatment				*P*-value	
Item	Ad	PF	PRRS+	SEM	Ad vs. others	PF vs. PRRS+
Morphology, μm						
Villus height	481	411	324	27.3	0.003	0.036
Crypt depth	293	228	243	19.2	0.017	0.588
V:C^2^	1.72	1.89	1.37	0.122	0.369	<0.001
Goblet cells/10,000 μm^2^	6.73	7.04	7.46	0.499	0.155	0.294

^1^ Pigs were either challenged with porcine respiratory and reproductive syndrome virus (PRRS+), PRRSV naïve and fed ad libitum (Ad), or PRRSV naïve and pair-fed to PRRS+ pigs intake (PF). Pigs were euthanized at days post inoculation (dpi) 17.

^2^V:C = Villus height:Crypt depth

### mRNA abundance of tight junction proteins

**Fig 1:**
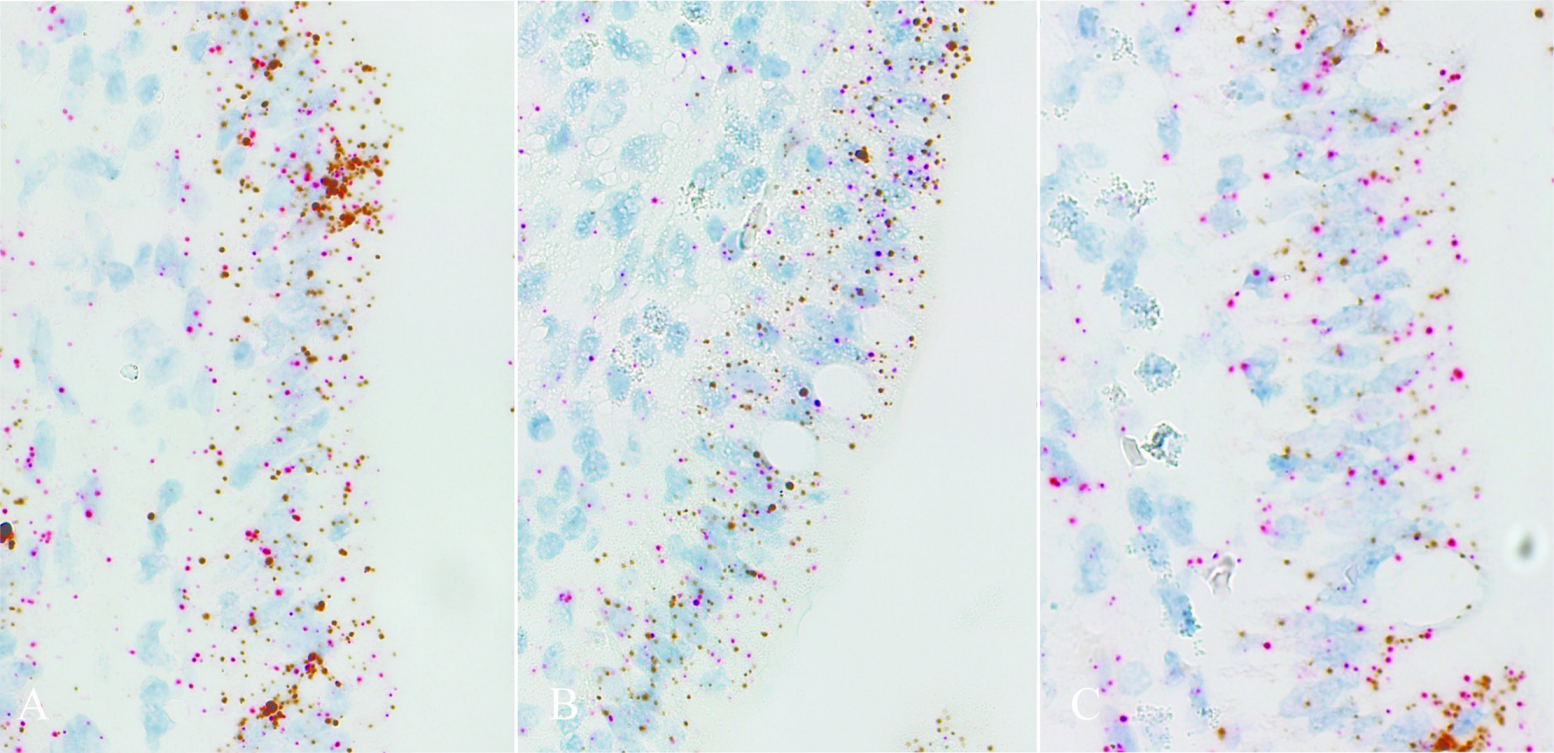
A-C. Representative RNAscope duplex for Occludin (brown) and ZO-1 (red). **A**) Pigs were either PRRSV naïve and fed ad libitum (Ad); **B**) PRRSV naïve and pair-fed to PRRS+ pigs intake (PF); or **C**) challenged with PRRSV (PRRS**+**).

Abundance of tight junction protein mRNA was measured via PCR (**Table 4**) and in-situ hybridization (**Table 5, Fig 1**). Claudin 2 mRNA abundance, measured via PCR, tended to be reduced in PRRS+ and PF pigs when compared with Ad pigs (*P* = 0.094) and was reduced in PRRS+ pigs when compared with PF pigs (*P* = 0.025). Claudin 3 mRNA abundance was reduced in both PRRS+ and PF pigs compared with Ad pigs (60%; *P* < 0.05) and did not differ from each other. Abundance of claudin 4 and occludin did not differ amongst treatments when measured via PCR (**Table 4**). Abundance of ZO-1 was reduced in PRRS+ pigs compared with PF pigs (*P* = 0.044) when measured via PCR (**Table 4**).

**Table 4:** Jejunum mRNA abundance as measured via PCR^1^.

	Treatment				*P*-value	
Item^2^	Ad	PF	PRRS+	SEM	Ad vs. others	PF vs. PRRS+
Claudin 2	1.45	1.34	0.69	0.209	0.094	0.025
Claudin 3	1.05	0.72	0.54	0.114	0.006	0.273
Claudin 4	0.91	1.21	0.76	0.223	0.778	0.133
Occludin	0.75	0.84	0.64	0.165	0.936	0.310
ZO-1	1.09	1.10	0.48	0.196	0.194	0.026
Glucose transporter 2	0.89	1.01	0.63	0.131	0.681	0.046
SGLT1	0.89	0.83	0.83	0.197	0.771	0.996
AMPK	0.94	0.94	0.38	0.336	0.204	0.474

^1^ Pigs were either challenged with porcine respiratory and reproductive syndrome virus (PRRS+), PRRSV naïve and fed ad libitum (Ad), or PRRSV naïve and pair-fed to PRRS+ pigs intake (PF). Pigs were euthanized at days post inoculation (dpi) 17.

^2^Gene abundances expressed as fold changes from Ad average (2^-ΔΔCt^)

**Table 5:** Gene abundance as measured by quantification of mRNA transcripts identified through RNAScope^®^ in-situ hybridization^1^.

	Treatment				*P*-value	
Item^2^	Ad	PF	PRRS+	SEM	Ad vs. others	PF vs. PRRS+
Claudin 4	4.89	3.73	3.15	0.443	0.003	0.222
Occludin	5.03	4.30	3.42	0.372	0.018	0.108
ZO-1	6.74	5.82	4.84	0.527	0.040	0.205
AMPK	2.18	1.77	1.50	0.105	<0.001	0.074

^1^ Pigs were either challenged with porcine respiratory and reproductive syndrome virus (PRRS+), PRRSV naïve and fed ad libitum (Ad), or PRRSV naïve and pair-fed to PRRS+ pigs intake (PF). Pigs were euthanized at days post inoculation (dpi) 17.

^2^Mean mRNA transcripts/cell

When measured via in-situ hybridization (**Table 5**), abundance of claudin 4 was reduced in PRRS+ and PF pigs compared with Ad pigs (*P* = 0.003) and did not differ from each other (**Table 5**). Similarly, occludin abundance was reduced in PRRS+ and PF pigs compared with Ad pigs (*P* = 0.018) and did not differ from another when measured via in-situ hybridization (**Table 5**). Abundance of ZO-1 was reduced in PRRS+ and PF pigs compared with Ad pigs (*P* = 0.040) and did not differ from another when measured via in-situ hybridization (**Table 5**).

### Tight junction protein abundance

**Fig 2:**
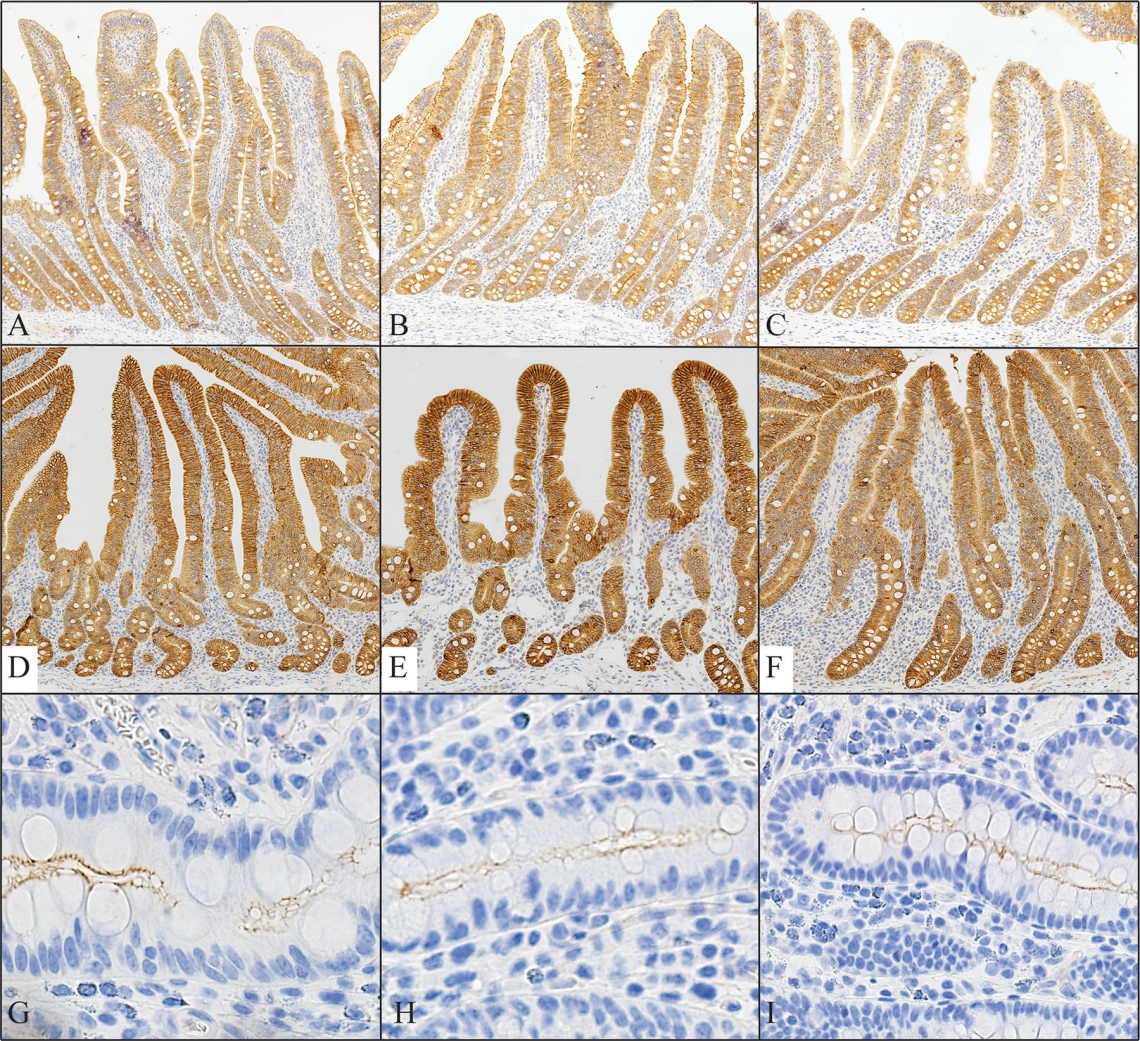
Representative immunohistochemistry images for Claudin 2 (**A-C**), Claudin 4 (**D-F**), and ZO-1 (**G-I**). Pigs were either PRRSV naïve and fed ad libitum (Ad: **A, D,** and **G**), PRRSV naïve and pair-fed to PRRS+ pigs intake (PF: **B, E,** and **H**), or challenged with PRRSV (PRRS+: **C, F,** and **I**). Pigs were euthanized at days post inoculation (dpi) 17. Staining was limited to the epithelial cells, with higher stain density at cell membranes.

Tight junction protein abundance was measured via Western blotting (**Table 6**) and immunohistochemistry (**Table 7, Fig 2**). Total abundance of claudin 4 and occludin did not differ amongst treatments when measured via Western blot (*P* > 0.10; **Table 6**). Measuring abundance via immunohistochemistry allows for evaluation of both total and localization of protein stain to determine the cellular location of these proteins. Total, cytoplasmic, and membrane abundance did not differ amongst treatments (*P* > 0.10; **Table 7**) for either claudin 2 or claudin 4. For ZO-1, cytoplasmic abundance tended to be increased in PRRS+ pigs when compared with PF pigs (*P* = 0.091). However, neither membrane nor total abundance of ZO-1 differed amongst treatments (*P* > 0.10).

**Table 6:** Jejunum protein abundance determined via Western blots^1^.

	Treatment				*P*-value	
Item^2^	Ad	PF	PRRS+	SEM	Ad vs. others	PF vs. PRRS+
Claudin 4	0.62	0.41	0.62	0.158	0.547	0.298
Occludin	0.37	0.28	0.30	0.107	0.518	0.920
pAMPK	1.12	1.12	1.06	0.288	0.947	0.885
AMPK	1.92	2.10	1.05	0.601	0.566	0.133
pAMPK:AMPK	0.99	0.83	1.03	0.251	0.853	0.587

^1^ Pigs were either challenged with porcine respiratory and reproductive syndrome virus (PRRS+), PRRSV naïve and fed ad libitum (Ad), or PRRSV naïve and pair-fed to PRRS+ pigs intake (PF). Pigs were euthanized at days post inoculation (dpi) 17.

^2^ Arbitrary units

**Table 7:** Jejunum protein abundance determined via immunohistochemistry staining^1^.

	Treatment				*P*-value	
Item^2^	Ad	PF	PRRS+	SEM	Ad vs. others	PF vs. PRRS+
Claudin 2						
Cytoplasmic	0.12	0.09	0.08	0.026	0.358	0.844
Membrane	0.12	0.09	0.08	0.028	0.337	0.818
Overall	0.06	0.05	0.05	0.013	0.302	0.893
Membrane:Cytoplasmic	0.99	0.93	0.05	0.025	0.092	0.578
Claudin 4						
Cytoplasmic	0.18	0.21	0.20	0.022	0.315	0.983
Membrane	0.20	0.23	0.22	0.026	0.378	0.886
Overall	0.12	0.14	0.14	0.014	0.399	0.964
Membrane:Cytoplasmic	1.12	1.12	1.10	0.011	0.416	0.466
ZO-1						
Cytoplasmic	0.0023	0.0012	0.0020	0.0025	0.126	0.091
Membrane	0.0035	0.0022	0.0025	0.0061	0.156	0.676
Overall	0.0023	0.0017	0.0020	0.0044	0.394	0.648
Membrane:Cytoplasmic	1.790	1.500	1.330	0.376	0.427	0.747

^1^ Pigs were either challenged with porcine respiratory and reproductive syndrome virus (PRRS+), PRRSV naïve and fed ad libitum (Ad), or PRRSV naïve and pair-fed to PRRS+ pigs intake (PF). Pigs were euthanized at days post inoculation (dpi) 17.

^2^Average positive optical density within the area of interest

### mRNA and protein abundance of nutrient transporters and energy sensors

mRNA abundance of the nutrient transporter glucose transporter 2 was reduced in PRRS+ pigs when compared with PF pigs (*P* = 0.046) when measured via PCR (**Table 4**). Conversely, abundance of sodium-dependent glucose co-transporter 1 did not differ amongst treatments when measured via PCR (*P* > 0.10). Similarly, mRNA abundance of the energy sensing protein AMPK did not differ (P > 0.10) when measured via PCR (**Table 4**). However, mean transcript abundance of AMPK was reduced in both PRRS+ and PF pigs compared with Ad pigs (25%; *P* < 0.001) and tended to be reduced in PRRS+ pigs compared with PF pigs (*P* = 0.074) when measured via in-situ hybridization (**Table 5**).

Protein abundance of AMPK and pAMPK did not differ amongst treatments (*P* > 0.10), nor was the ratio of phosphorylated to total AMPK altered by treatment when measured via Western blotting (**Table 6**).

## Discussion

Adverse health events and the accompanying hypophagia results in economically significant losses in pig growth performance worldwide. However, it is unclear how an adverse health event such as PRRSV challenge directly, or indirectly via hypophagia, impacts small intestinal function and integrity [8]. Moreover, little is known about how much disease hypophagia contributes to attenuated pig performance and modulations in small intestinal function and integrity. In order to investigate this, a 17 day PRRSV challenge study was conducted in growing pigs. In addition, a treatment group comprising of PRRSV naïve pigs pair-fed to PRRSV challenged pigs’ daily feed intake was utilized to investigate the extent to which disease hypophagia modulates jejunum function and integrity.

In PRRSV challenge studies of similar duration, voluntary feed intake is commonly reduced 25–30% [18, 34, 35], although reductions in feed intake of up to 40% have been reported [36]. In the current study, feed intake was reduced 45% in PRRS+ pigs, a more severe reduction than what has been reported. This is likely due to the age of pig and/or virulence of PRRSV strain utilized [37]. We also reported herein that PRRS+ pigs had accompanying reductions in growth rates. As such, maintenance energy calculations based off NRC [38] requirements demonstrated both PRRS+ and pair-fed pigs consumed more feed than was required for maintenance of body weight (1.6 and 1.7 times maintenance energy requirements, respectively), however this does not account for the additional energy expenditure required for the immune response. Thus, the caloric restriction model in pair-fed pigs was not severe enough to induce starvation-like changes to the intestinal epithelium but was severe enough to induce any nutrient sparing adaptive responses that may have occurred during the 17 day test period.

The absorptive enterocytes of the small intestinal epithelium contain a variety of transporters and transport mechanisms to efficiently absorb dietary nutrients from the lumen. However, these absorptive epithelial cells undergo constant migration and turnover, an energetically demanding process. In starvation studies utilizing mice [39] and rats [12], both epithelial cell migration and turnover are reduced, suggesting that the intestine reduces cellular turnover to spare energy in a time of nutrient scarcity. This is accompanied by a reduction in jejunal villus height and crypt depth [12]. Although reducing cellular turnover spares energy, it also reduces the absorptive area of the intestinal epithelium, making potential caloric interventions less effective. In previous work examining PRRSV infected pigs, our laboratory reported no alteration in jejunal morphology at dpi 21 [40]. In contrast, at dpi 7 PRRSV infected pigs have been reported to have marked reductions in jejunal villus height and crypt depth [36]. These discrepancies may be due to differences in the PRRSV pathogenicity or feed intake. Herein, we report that PRRS+ and pair-fed pigs had reduced jejunal villus height and crypt depth at dpi 17 compared with Ad pigs. While changes to crypt depth were similar in PRRS+ and pair-fed pigs, reductions to villus height were more severe in PRRS+ pigs, suggesting that caloric intake partially, but not fully, explains morphology changes. The additional reduction in villus height in PRRS+ pigs may be a direct result of PRRSV itself or may be a function of the additional energy required for the immune response, which would put PRRS+ pigs at an energy deficit compared with pair-fed pigs. Regardless, it appears as though restoring feed intake could at least partially restore epithelial cell turnover and morphology of the digestive tract.

Although morphology is a good marker of absorptive area of the intestine, digestive and absorptive enzymes are a more direct measure of digestive efficiency. Digestion of disaccharides such as sucrose, lactose, and maltose in the small intestine occurs via the brush border disaccharidases sucrase, lactase, and maltase, respectively. In neonatal pigs feed restricted by 60% for 30 d, activities of jejunal disaccharidases were decreased when expressed on an area of tissue basis, but were reported to be upregulated when expressed on a protein basis [11]. These data suggest that total brush border enzyme abundance may be reduced, but activity is unhindered. In the current experiment, activities of the disaccharidases (sucrase, lactase, maltase) were not different from Ad pigs as a result of PRRSV or pair-feeding. However, the activities of all 3 brush border enzymes were reduced in PRRS+ pigs when compared with pair-fed pigs. Although little is known about the regulation of disaccharidase activity, it is possible that these differences are due to disparities in the nature of the feed restriction model (voluntary vs. involuntary).

Following enzymatic degradation, luminal glucose uptake into the enterocyte occurs actively via sodium-dependent glucose co-transporter 1 and passively via glucose transporter 5 [41]. In the current experiment, *ex vivo* active transport of both glucose and glutamine were assessed in modified Ussing chambers. Both glucose and glutamine active transport were increased in PRRS+ pigs compared with Ad pigs. This is in agreement with previous work in our laboratory using health compromised pigs, in which increased *ex vivo* active nutrient transport has been observed in 21 day PRRSV challenged pigs [40], as well as bacterially challenged [42], lipopolysaccharide challenged [43], and heat stressed [3] pigs. We have postulated that these changes were partially driven by a reduction in feed intake and possibly a host response to nutrient and energy scarcity. The utilization of the pair-feeding model was able to demonstrate that increased active nutrient transport is partially driven by a reduction in feed intake, as pair-fed pigs had active nutrient transport values intermediate that of PRRS+ and Ad pigs. The additional increase in active nutrient transport in PRRS+ pigs may be an adaptive mechanism to support changes in metabolism that occur during immune stimulation to reflect heightened glucose demand [44, 45]. Active glucose transport is driven by a sodium gradient generated by basolateral Na+/K+ ATPase pumps [46]. Often, increases in *ex vivo* glucose transport are accompanied by increases in activity of Na+/K+ ATPase pumps [3, 40]. However, in the current experiment jejunum Na+/K+ ATPase activity was slightly reduced by PRRSV and pair-feeding. Although the reasoning for this is unclear, inconsistencies may be due to differences in duration or severity of the stressor challenge. After uptake from the lumen, some glucose is utilized by the enterocyte for ATP generation, however most of the glucose is transferred into the bloodstream via the passive basolateral transporter glucose transporter 2. We observed no change in mRNA abundance of glucose transporter 2 between Ad pigs and PRRS or pair-fed pigs, which is in agreement with previous PRRSV challenge studies [40]. Taken together, it appears that neither PRRSV challenge nor nutrient restriction caused a significant reduction in digestive or absorptive activity of the intestinal epithelium.

In addition to its role in digestion and nutrient uptake, the intestinal epithelium acts as a barrier against toxins and harmful microorganisms. Critical to the integrity of this barrier are goblet cells and the tight junction complexes. Goblet cells produce the primary defense mechanism, a mucus layer which physically prevents bacterial penetration [47]. Stressors such as underfeeding [11] and PEDV infection [48] have been shown to deplete goblet cells, thus reducing mucin production and leaving pigs more susceptible to further bacterial infection. However, in the current experiment, no difference in goblet cell count was observed. These data are in agreement with Schweer et al. [40], who observed no difference in jejunal mucin protein abundance resulting from PRRSV challenge.

Tight junction complexes form the secondary defense barrier, a carefully regulated selectively permeable seal between epithelial cells. Reduced integrity of the tight junction barrier is associated with lower transepithelial resistance and a higher permeability to macromolecules [49]. Stress induced changes in intestinal integrity has been well documented in pigs, particularly regarding weaning stress [see 5 for review] and heat stress [3, 4, 50]. However, mRNA and protein abundance and localization of tight junction proteins has been poorly characterized in post-weaned pigs under stressors such as disease challenge. As mRNA abundance is the most commonly reported marker of intestinal integrity in pig studies but does not indicate if protein membrane abundance or total abundance is altered, both mRNA and protein analysis approaches were chosen in order to examine if these measures correlate under PRRSV challenge. In the current study, we observed reductions in jejunum transepithelial resistance as a result of PRRSV challenge, but no significant change in macromolecule permeability compared with Ad pigs, although high variation in these measures was reported. Interestingly, these changes were similar to those of pair-fed pigs, suggesting that caloric and nutrient restriction explains the reduction in jejunal integrity reported herein. These data somewhat agree with that of Jacobi et al. [51], who observed no significant changes in intestinal transepithelial resistance or mannitol permeability in suckling pigs after 3 days of 50% caloric restriction. These authors did report numerical reductions in transepithelial resistance due to caloric restriction, but not mannitol permeability [51]. This suggests that under a calorie restricted state the passage of small ions is altered, but not the integrity of tight junctions. In contrast, protein-energy malnourishment in pigs (50% caloric restriction and protein deficient diet), did result in greater FD4 permeability [52, 53]. These inconsistencies may be due to the specific nature of the malnutrition model. In studies where macromolecule permeability was increased [52, 53], pigs were both energy restricted and fed protein deficient diets, whereas in studies where no change in macromolecule permeability was observed [51; the current study], pigs were energy restricted but fed diets that met their specific nutrient requirements and should not have been in a caloric deficit severe enough to become deficient in specific nutrients such as protein, which is used preferentially and disproportionately by the intestine for maintenance [54]. In support of this, we observed no difference in the phosphorylation (activation) of the energy sensing protein AMPK in the jejunum. Activation of AMPK is observed in pig jejunum tissue during chronic lipopolysaccharide administration [55], and activated AMPK partially mediates reductions in intestinal integrity during interferon-γ induced inflammation via reducing occludin and ZO-1 protein abundance [56]. Thus, preservation of energy homeostasis may have been responsible for maintenance of tight junction complexes.

To confirm our hypothesis that tight junction complex integrity was maintained, the abundance and localization of tight junction proteins was assessed, as these proteins are believed to play the greatest role in intestinal integrity [2]. The transmembrane protein occludin possesses adhesive properties [57] to assist with seal formation. The transmembrane claudins can be classified as either sealing, such as claudin 4, or pore forming, such as claudin 2 [49]. Regardless, stability of these transmembrane proteins are reliant on interactions with intracellular proteins such as ZO-1 [58]. As tight junction proteins can be internalized under stress independently of cell energy status [56, 59], we also evaluated cellular location (membrane or cytoplasm) via immunohistochemistry, to better determine the integrity of the complexes. In confirmation, we observed no change to the cellular location of any of the tight junction proteins evaluated, similar to what has been observed with cellular location of occludin in calorie restricted piglets [51] and with ZO-1 and claudin-1 in malnourished mice [60]. However, we did observe decreased mRNA abundance of several tight junction proteins when quantified through both PCR and in-situ hybridization. Decreased mRNA abundance of tight junctions proteins was not observed in less severely PRRSV challenged pigs [40], but has been documented in protein-energy restricted pigs [52]. The reduced mRNA abundance documented herein may be the result of reduced epithelial cell turnover, which allows the body to minimize the energetic output of the gastrointestinal system, whilst remaining impermeable to pathogens and equipped to absorb nutrients once the pig’s appetite has returned [12, 61]. A reduction in the rate of epithelial cell turnover would result in maintenance of epithelial cell protein abundance but would reduce need for mRNA synthesis. This could explain why tight junction mRNA abundance was decreased, but no change was observed in tight junction protein abundance or the integrity of the tight junctions to permeation by macromolecules. It also suggests that in studies involving long term biological adaptation to disease, evaluating mRNA abundance may not explain what changes are occurring at a protein or protein function level, as these do not necessarily correlate well during adaptive periods such as starvation [62]. However, as pigs were only euthanized at one time point, it is also possible that given more time, protein abundances would reflect the differences observed in mRNA abundance.

In conclusion, this study demonstrates that most of the changes that occur to intestinal structure, function, and integrity during a systemic PRRSV challenge are partially a function of hypophagia. Further, during periods of disease induced hypophagia, jejunal digestive machinery retains its ability to digest and absorb nutrients. Finally, although long term adaptation to systemic PRRSV challenge and caloric restriction reduces intestinal integrity based on transepithelial resistance, it does not appear to increase macromolecule permeability or reduce the integrity (abundance and location) of tight junction protein complexes.

## Supporting information

S1 Table Primer sequences. (DOCX)

S1 Raw images Western blot image file. (DOCX)

S1 Dataset Raw data from which manuscript was written. (XLSX)
