# Supplementary material for: Impact of viral disease hypophagia on pig jejunal function and integrity
Source: PLoS One. 2020 Jan 7;15(1):e0227265. doi: 10.1371/journal.pone.0227265 (PMC6946155; doi:10.1371/journal.pone.0227265)
Supplement: S1 Table — (DOCX) [file pone.0227265.s001.docx]

| **Gene** | **Sense (5'-3') - forward** | **Antisense (5'-3') - reverse** |
| --- | --- | --- |
| ACTB | CCAACATTGGTTATGGGAGCAA | GGAAGAGACGTTGTGAGCAA |
| AMPK | GTATGCTGGTCCAGAGG | AAAGGCTAATCACAGAAGG |
| CLDN2 | AGGCCTCCTGGGCTTCAT | GGAGTAGAAGTCCCGCAGGAT |
| CLDN3 | TTGCATCCGAGACCAGTCC | AGCTGGGGAGGGTGACA |
| CLDN4 | GTATCATCCTGGCCGTGCTA | TTGGCGCTCTCATCATCCA |
| GLUT2 | TCATCAGCTGGCCATTGTCA | GCTCATGATTGCCCAGGAGAA |
| OCLN | TCGTCCAACGGGAAAGTGAA | ATCAGTGGAAGTTCCTGAACCA |
| SLC5A1 | GGCTGTTCCAACATTGCCTA | CAACATGACCGACAGCATCA |
| ZO1 | AAGCCCTAAGTTCAATCACAATCT | ATCAAACTCAGGAGGCGGC |

**S1 Table**. Primer sequences
