# Supplementary material for: Impact of viral disease hypophagia on pig jejunal function and integrity
Source: PLoS One. 2020 Jan 7;15(1):e0227265. doi: 10.1371/journal.pone.0227265 (PMC6946155; doi:10.1371/journal.pone.0227265)
Supplement: S1 Raw images — (DOCX) [file pone.0227265.s002.docx]

1. Raw, uncropped, and unadjusted blot for occludin


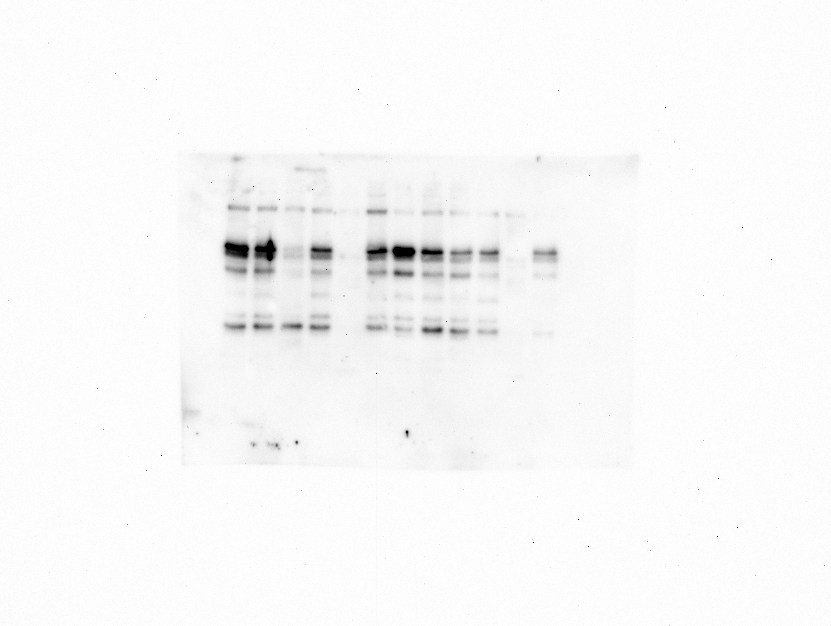

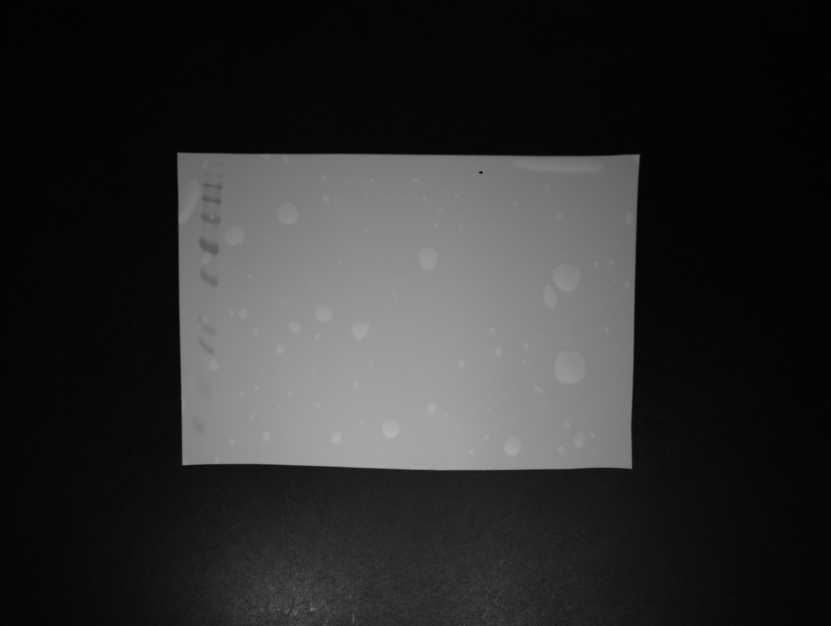


1 2 3 4 5 6 7 8 9 10 11 12

Occludin (65 kDa)

75 kDa

50 kDa

25 kDa

2 kDa

**Notes:**

Results shown are representative images

Capture method – chemiluminescence, 2 m 40 s exposure

**Lane key:**

1. Ad, control sample (run on all gels)
2. PF
3. PRRS+
4. Ad
5. PF
6. PRRS+
7. Ad
8. PF
9. PRRS+
10. Ad
11. PF
12. PRRS+
13.
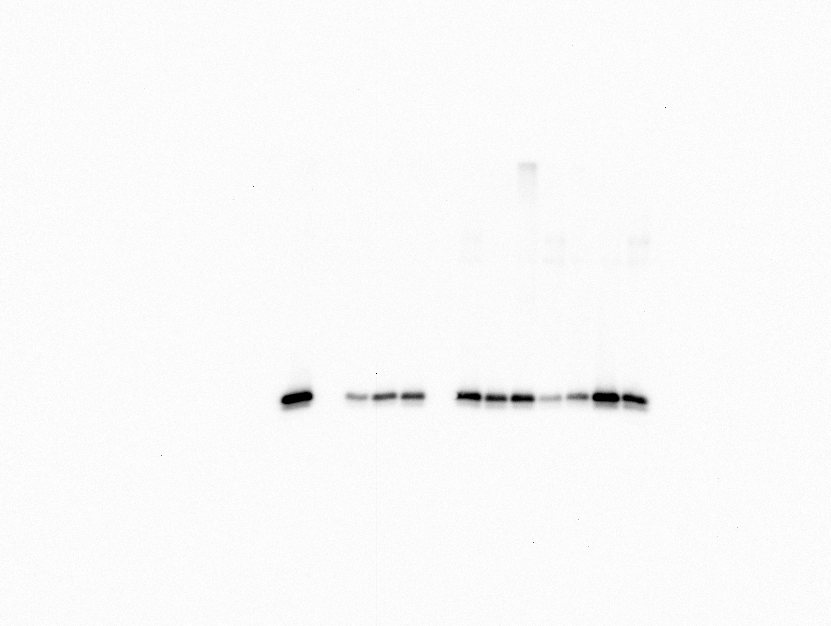

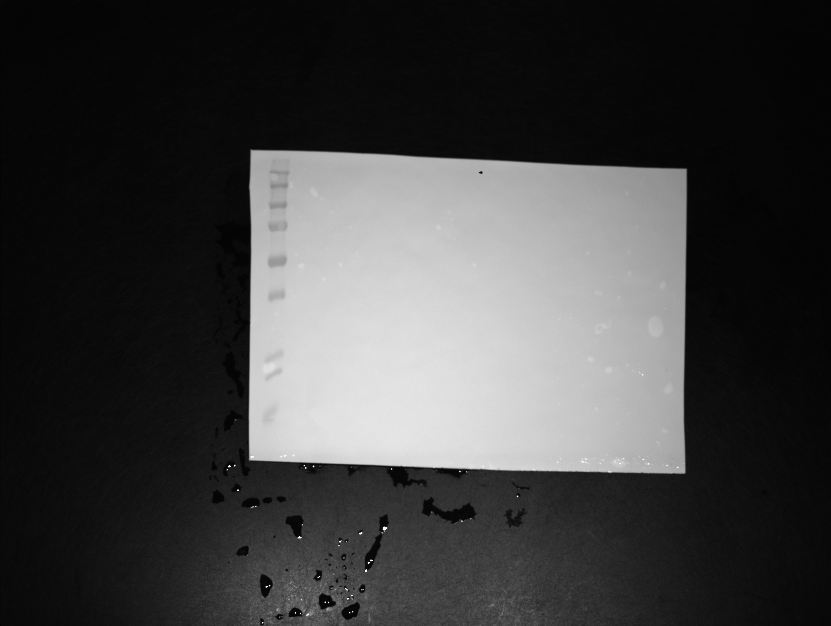
Raw, uncropped, and unadjusted blot for claudin 4

75 kDa

50 kDa

25 kDa

Claudin 4 (22-kDa)

2 kDa

1 2 3 4 5 6 7 8 9 10 11 12 13

**Notes:**

Results shown are representative images

Capture method – chemiluminescence, 40 s exposure

**Lane key:**

1. Ad, control sample (run on all gels)
2. Ad
3. PF
4. PRRS+
5. Ad
6. PF
7. PRRS+
8. Ad
9. PF
10. PRRS+
11. Ad
12. PF
13. PRRS+
14.
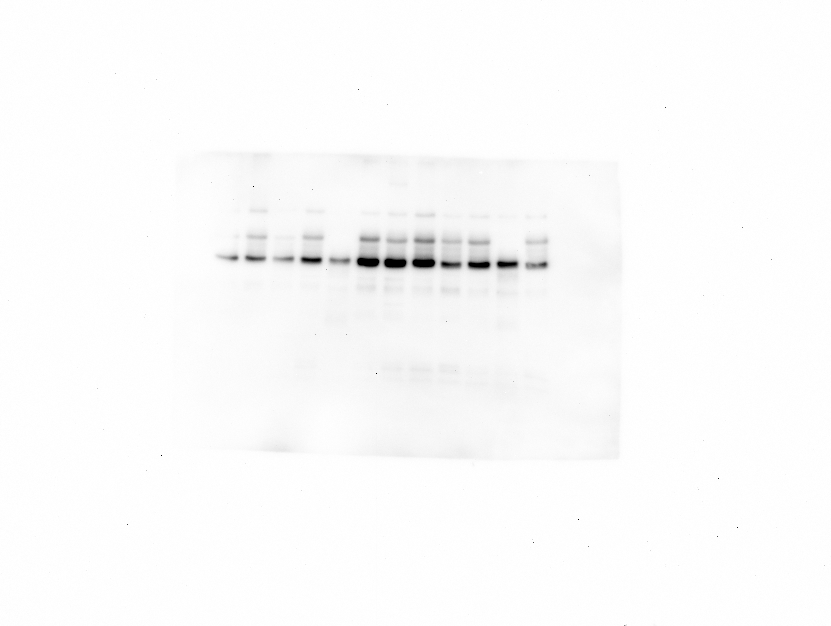

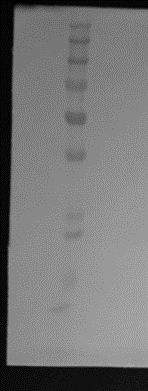
Raw, uncropped, and unadjusted blot for AMPKα

1 2 3 4 5 6 7 8 9 10 11 12

2 kDa

25 kDa

50 kDa

75 kDa

AMPKα (62-kDa)

**Notes:**

Results shown are representative images

Capture method – chemiluminescence, 2 m 40 s exposure

**Lane key:**

1. Ad, control sample (run on all gels)
2. PF
3. PRRS+
4. Ad
5. PF
6. PRRS+
7. Ad
8. PF
9. PRRS+
10. Ad
11. PF
12. PRRS+
13.
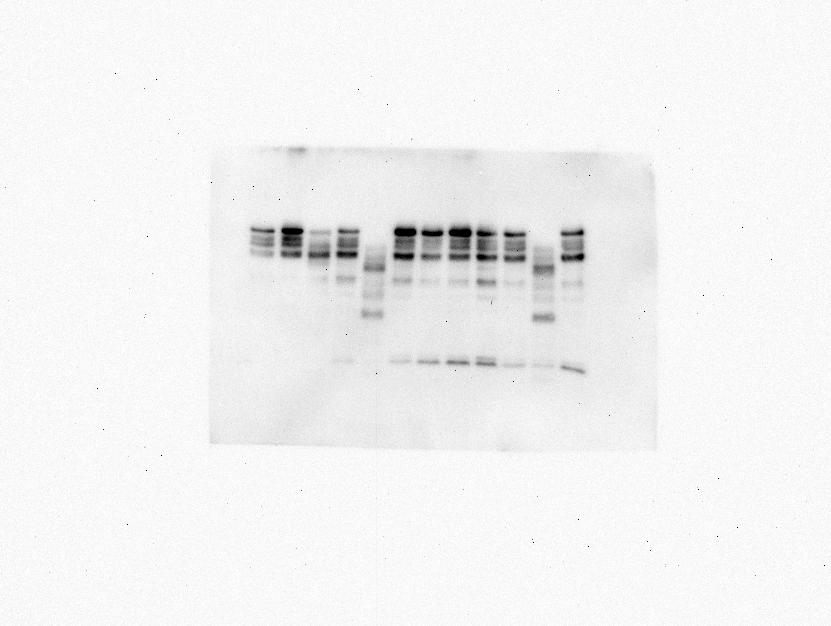

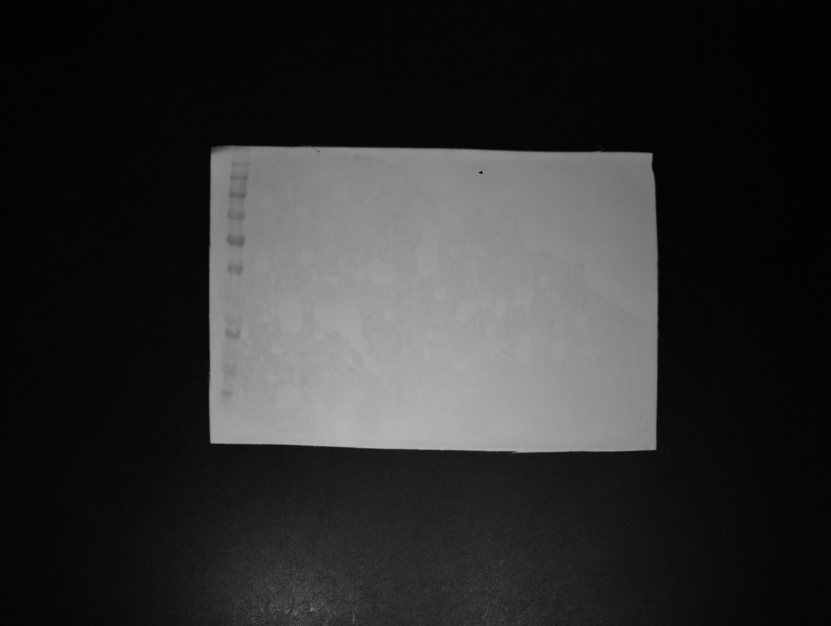
Raw, uncropped, and unadjusted blot for pAMPKα

1 2 3 4 5 6 7 8 9 10 11 12

2 kDa

25 kDa

50 kDa

75 kDa

AMPKα Thr^172^ (62-kDa)

**Notes:**

Results shown are representative images

Capture method – chemiluminescence, 2 m 40 s exposure

**Lane key:**

1. Ad, control sample (run on all gels)
2. PF
3. PRRS+
4. Ad
5. PF
6. PRRS+
7. Ad
8. PF
9. PRRS+
10. Ad
11. PF
12. PRRS+
